# Co-localization and regulation of basic fibroblast growth factor and arginine vasopressin in neuroendocrine cells of the rat and human brain

**DOI:** 10.1186/1743-8454-7-13

**Published:** 2010-08-13

**Authors:** Ana M Gonzalez, William M Taylor, Conrad E Johanson, Joan C King, Wendy E Leadbeater, Edward G Stopa, Andrew Baird

**Affiliations:** 1 School of Clinical and Experimental Medicine, College of Medical and Dental Sciences, University of Birmingham, Birmingham B15 2TT, UK; 2 Departments of Pathology and Neurosurgery, Rhode Island Hospital, Brown Medical School, Providence, RI 02903, USA; 3 Human Brain Tissue Resource Center, McLean Hospital/Harvard Medical School, Belmont, MA 02178, USA; 4 Department of Anatomy and Cellular Biology, Tufts University School of Medicine, Boston, MA 02111, USA; 5 Department of Surgery, UCSD School of Medicine, San Diego, California, 92103, USA

## Abstract

**Background:**

Adult rat hypothalamo-pituitary axis and choroid plexus are rich in basic fibroblast growth factor (FGF2) which likely has a role in fluid homeostasis. Towards this end, we characterized the distribution and modulation of FGF2 in the human and rat central nervous system. To ascertain a functional link between arginine vasopressin (AVP) and FGF2, a rat model of chronic dehydration was used to test the hypothesis that FGF2 expression, like that of AVP, is altered by perturbed fluid balance.

**Methods:**

Immunohistochemistry and confocal microscopy were used to examine the distribution of FGF2 and AVP neuropeptides in the normal human brain. In order to assess effects of chronic dehydration, Sprague-Dawley rats were water deprived for 3 days. AVP neuropeptide expression and changes in FGF2 distribution in the brain, neural lobe of the pituitary and kidney were assessed by immunohistochemistry, and western blotting (FGF2 isoforms).

**Results:**

In human hypothalamus, FGF2 and AVP were co-localized in the cytoplasm of supraoptic and paraventricular magnocellular neurons and axonal processes. Immunoreactive FGF2 was associated with small granular structures distributed throughout neuronal cytoplasm. Neurohypophysial FGF2 immunostaining was found in axonal processes, pituicytes and Herring bodies. Following chronic dehydration in rats, there was substantially-enhanced FGF2 staining in basement membranes underlying blood vessels, pituicytes and other glia. This accompanied remodeling of extracellular matrix. Western blot data revealed that dehydration increased expression of the hypothalamic FGF2 isoforms of ca. 18, 23 and 24 kDa. In lateral ventricle choroid plexus of dehydrated rats, FGF2 expression was augmented in the epithelium (Ab773 as immunomarker) but reduced interstitially (Ab106 immunostaining).

**Conclusions:**

Dehydration altered FGF2 expression patterns in AVP-containing magnocellular neurons and neurohypophysis, as well as in choroid plexus epithelium. This supports the involvement of centrally-synthesized FGF2, putatively coupled to that of AVP, in homeostatic mechanisms that regulate fluid balance.

## Background

Fluid homeostasis in the brain and periphery is mediated by peptides synthesized in extrahypothalamic and hypothalamic regions, respectively, of the CNS. In regard to brain water balance, the arginine vasopressin (AVP) expressed in choroid plexus epithelium (parenchymal cells) exerts an autocrine effect on cerebrospinal fluid (CSF) secretion [1-3]. CSF derived from choroid plexus is the primary determinant of brain extracellular fluid volume and composition [4]. Such CSF formation is controlled partly by vasopressinergic (V1) inhibitory effects on choroidal blood flow [5-7] and ion transport [8]. Choroid epithelial ultrastructure is also transformed by AVP, again suggesting a peptide-induced reduction in fluid movement at CNS transport interfaces [9]. On the other hand, peripheral water balance is regulated by hypothalamic AVP which is delivered to the neurohypophysis eventually to adjust renal ion transport and thus water balance.

Although basic FGF (FGF2) and its receptors [10-12] are known to be expressed in choroid plexus [13,14], their multiple functions await further elucidation. Growth factors, like neuropeptides, exert acute actions on blood flow and transport in various epithelia. For example, FGF2 infusion alters tissue perfusion by changing vessel diameter [15]. Moreover, exogenously administered FGF2 and AVP reduce CSF formation rate [7,16] and induce the dark choroid epithelial cells implicated in neuroendocrine regulation of water balance [8,9,17]. Because FGF high affinity receptors (FGFR) co-localize with AVP in parenchymal cells of choroid plexus [18], it is tempting to postulate a functional symmetry, perhaps coupling, that links FGF2, AVP and CNS water balance.

FGF2 and AVP are also expressed in the hypothalamus and hypophysis [14,19]. But while stimuli that activate magnocellular neurons in the paraventricular (PVN) and supraoptic nucleus (SON) enhance secretion of AVP into blood and CSF [20], FGF2 function in the hypothalamo-hypophyseal system is less understood. Still, the prominent distribution of FGF2 and its receptors within SON, PVN, median eminence and neurohypophysis, as well as choroid plexus, implies a role for FGF2 in ion/water homeostasis [14,18,19,21-23]. To this end, the more recent observations intimating a functional link between FGF2 secretion and the ion-transporting Na^+^-K^+ ^ATPase [24,25] strongly supports involvement of FGF2 in water homeostasis. FGF2 and AVP co-localize in epithelial cells of the choroid plexus. Therefore we postulated that these peptides also occur together in certain neuroendocrine cells of the hypothalamus, another region involved in fluid homeostasis.

The aim of these studies was to characterize the distribution and modulation of FGF2 in the human and rat CNS. In the findings presented here, we analyzed the distribution of FGF2 and AVP immunoreactivity in the SON, PVN, and neurohypophysis in human specimens obtained at autopsy. Confocal microscopy clearly revealed co-localization of FGF2 and AVP in SON as well as PVN magnocellular neurons. Moreover, given the increased expression of FGF2 in hypothalamus and choroid plexus now being reported in a rat model of disrupted water balance, our findings fit the model that central FGF systems help to mediate fluid homeostasis *in vivo *[19,23].

## Methods

### Human studies

**Figure 1:**
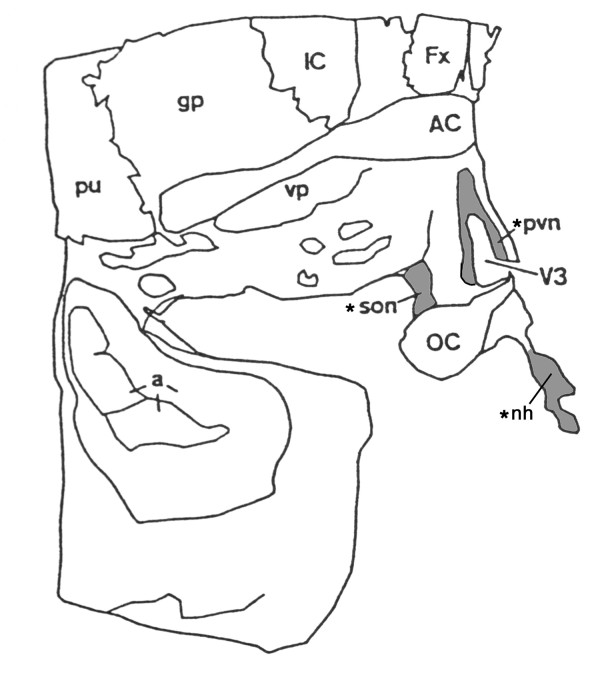
**Diagram depicting sampling sites in human brain**: Shaded areas are regions photographed*: supraoptic nucleus (SON, son), paraventricular nucleus (PVN, pvn) and neurohypophysis (nh). Adjacent sites include third ventricle (V3) and optic chiasm (OC). Choroid plexus tissue is attached to the roof of the 3rd ventricle and on the floor of the lateral ventricle (not shown) that is located above the fornix (Fx). IC, intermediate capsule; gp, globus pallidus; pu, putamen; AC, anterior commissure; vp, ventral pallidum; a, amygdala.

All human tissue samples were obtained with the approval of the Local Research and Ethics Committee. Female and male brain specimens (n = 5) were obtained within 4-18 h of death, and subjected to neuropathologic examination to exclude CNS abnormalities. The hypothalamus was dissected en-bloc and fixed in 4% formaldehyde. Specimens were immersed in 30% sucrose and snap frozen in liquid N_2 _for storage at -80°C. Coronal sections were cut on a freezing microtome at 8 μm and mounted on positive-charged slides. Fig. 1 illustrates the anatomical regions used in the current studies and from which representative photographs were obtained.

Immunohistochemical procedures used specific polyclonal antibodies raised against human FGF2 (Ab773) and AVP (Immunostar, Hudson, WI, USA). This antibody has been shown not to cross react with oxytocin. Double fluorescence labeling was performed sequentially. Briefly, 8-μm slide-mounted sections were washed in 0.05 M TBS buffer pH 7.6, then pre-treated with 3% H_2_O_2 _in methanol, 1% milk, 1.6% ammonium acetate, 0.1% normal goat serum (Jackson ImmunoResearch, West Grove, PA, USA) in 0.05 M TBS and 0.1% Triton X-100 before incubation with the rabbit anti-FGF2 antibody (Ab773, 1/1000) for 24 h at 4°C. Sections were rinsed thoroughly with 0.05M TBS pH7.6 containing 0.2% Triton and then incubated with rhodamine-tagged goat anti rabbit (Jackson ImmunoResearch) for 3 h at 4°C. Slides were rinsed thoroughly with 0.05M TBS pH7.6 containing 0.2% Triton, incubated with rabbit anti-vasopressin antibody (1/1000) for 24 h at 4°C, rinsed and incubated with fluorescein-tagged goat anti rabbit (Jackson ImmunoResearch) for 3 h at 4°C. Slides were finally rinsed in TBS buffer, followed by distilled water, coverslipped and stored in the dark.

The specificity of the antibodies used for double staining was assessed by the following controls. First, to test for cross reaction of the primary antibodies, sections were adsorbed with excess purified human FGF2 in diluted antiserum. FGF2 immunoreactivity disappeared and AVP immunoreactivity was unchanged, indicating that the FGF2 antibody did not recognize AVP (not shown). Then the specificity of the secondary linking antibodies was tested by omitting primary antibodies in the respective experiments. Such tests indicated that each secondary antibody did not cross-react with either the primary or the linking antibody that visualized the other protein. Antibody specificity was also confirmed from differential distribution of FGF2 and AVP staining in various regions of brain (not shown).

Fluorescent preparations were examined by a Nikon Confocal Microscope and a krypton/argon laser with a 580-nm long-pass dichroic filter. Tissues were excited at wavelengths of 488 or 568 nm and images obtained with barrier filters of 515 or 590nm. Images were collected as single pairs or sets of sequential images. Pixel dimensions (x, y) were 0.56 (20x) and 0.09 μm (100x, oil). The z dimension either matched the x, y pixel dimension or was larger, i.e. spaced farther apart to generate a series over a larger distance. Images were scanned on the two channels (red, LRSC and green, FITC) and merged to produce a single profile. In this mode, all regions exhibiting co-localization of red and green emitter produced yellow fluorescence. Relationships between immunoreactive FGF2- and AVP-containing elements were visualized in 2 and 3 dimensions using InterVision software (Thermo Noran, Inc., Madison, WI, USA).

### Rat Studies

All animal procedures were carried out with strict adherence to guidelines in the NIH Guide for the Care and Use of Laboratory Animals and with the approval of the Local Research and Ethics Committee. Adult male Sprague-Dawley rats (200 to 250g) were housed under normal laboratory conditions (12-h light/dark cycles) with food and water *ad libitum *for one week prior to experimentation. Rats were divided into two groups of 8 rats. In the water-deprivation group, no water was provided for 72h. Control rats received tap water. Both groups accessed food *ad libitum*. Prior to sacrifice, animals were anesthetized by pentobarbitone overdose (125 mg/kg, i.p), perfused transcardially with physiological saline followed by 4% formaldehyde in PBS. Brains were extricated, post-fixed overnight, dehydrated and embedded in paraffin.

For demonstration of FGF2 immunoreactivity, two rabbit polyclonal antibodies were used: (1) Ab773, an antibody raised against amino acids 1-24 of bovine FGF2 (1-146) with high affinity for extracellular FGF2 [26]. This antibody has broad cross-reactivity with FGF2 from several animal species; and (2) Ab106, an antibody raised against amino acids 1-23 of rat FGF2 (1-146). Ab106 recognizes rat FGF2 and has high affinity for intracellular FGF2. A purified IgG antibody fraction was prepared by passage over a protein A sepharose column (Amersham Pharmacia Biotech, Piscataway, NJ, USA). Staining specificity was verified by immunostaining sections with the antibody in the presence of the antigenic peptide or the eluant from the affinity column used to purify the antibody. For the rat studies, vasopressin immunoreactivity was demonstrated using a rabbit polyclonal antibody against AVP (Chemicon, Millipore Billerica, MA, USA). This antibody recognizes vasopressin and has < 1% cross-reactivity with oxytocin. Immunostaining was done with a modified avidin-biotin complex technique described previously [19]. Endogenous peroxidase was quenched by treating the 8-μm section with 0.5% H_2_O_2 _in PBS for 30 min. Tissue sections were rinsed and incubated in 1.5% normal goat serum (Jackson ImmunoResearch), and then incubated at 4°C overnight with Ab773 or Ab106 diluted in PBS containing 0.3% triton and 5% bovine serum albumin (Jackson ImmunoResearch). Sections were then incubated with goat biotinylated anti-rabbit antibody (Vector, Burlingame, CA, USA), followed by an avidin-biotin-peroxidase complex (Vector). Finally, the sections were treated with 0.5% diaminobenzidine (DAB, Sigma, St. Louis, MO, USA) in PBS containing 0.01% H_2_O_2_. DAB-treated sections were counterstained with hematoxylin, rinsed, dehydrated, and protected with coverslips. Alternatively, tissue sections were incubated for 30 min with a fluorescein-labeled donkey anti-rabbit antibody (Jackson ImmunoResearch), rinsed, protected with coverslips and observed under an epifluorescence microscope.

Immunoblotting for FGF2 was performed on rat hypothalamus (anterior, medial, dorsal and posterior), as well as on heart (atria plus ventricles) and kidney (cortex plus medulla), in order to compare levels of FGF2 protein expression in neural vs. non-neural tissues. Western blot procedures [25] were performed with some modifications: the hypothalami and other tissues were homogenized in iced extraction buffer [10 mM Tris pH 7.4, containing 2M NaCl, 1 mM EGTA, 1 mM EDTA, 0.4 mM PMSF (Calbiochem, EMD Chemicals, Gibbstown, NJ, USA), 5 μg/ml pepstatin A (MP Biomedicals, Irvine, CA, USA), 5 μg/ml aprotinin (Sigma), and 5 μg/ml leupeptin (MP Biomedicals)], centrifuged at 14,000 rpm at 4°C and the pellet re-suspended in 10 mM Tris, pH 7.4. Protein concentrations were determined by the BCA protein assay reagent (Thermo Scientific, Rockford, IL, USA). Equal amounts of protein (500 μg) from each sample were incubated for 16 h at 4°C with pre-washed heparin-Sepharose CL-6B (Pharmacia Sweden). Beads were then washed with 10 mM Tris buffer pH 7.4 and re-suspended in 50 μl of Laemli's buffer (Sigma). Samples were heated at 100°C for 3 min and the supernatant electrophoresed on 18% SDS-PAGE and electroblotted onto nitrocellulose. The membrane was incubated in 10 mM Tris buffer pH 7.4 containing 5% non-fat milk (NFM/TBS) for 48 h at 4°C, and then incubated with rabbit anti-rat FGF2 (Ab106) diluted in NFM/TBS for 1h at room temperature, rinsed and incubated with ^125^I-Protein A (2 μC/10 ml) (GE Healthcare, Amersham, Piscataway, NJ, USA) in NFM/TBS. Signals were detected by autoradiography. Immunoblotting was repeated three times with different preparations of tissue extracts.

## Results

### FGF2 and AVP immunoreactivity in human paraventricular nucleus

**Figure 2:**
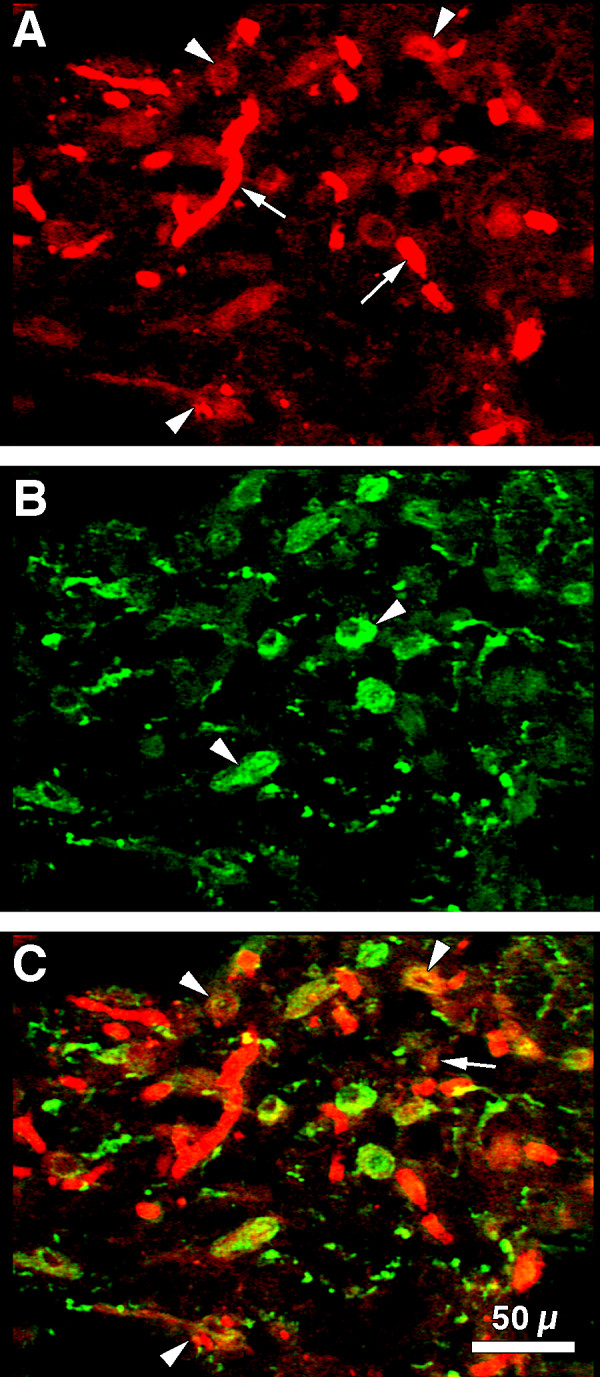
**Co-localization of FGF2 and AVP in human brain: **(A) FGF2 immunoreactivity was found within human magnocellular neurons and their processes (arrowheads). FGF2 immunostain was observed within endothelium of cerebral vessels (arrows) and in astrocytes and their processes (bar = 50 μm). (B) Note AVP immunoreactivity within neuronal perikarya and processes (arrowheads) of magnocellular neurons in the PVN of adult human hypothalamus. Axons are clearly distinguishable because their 'beaded' structure (bar = 50 μm). (C) Composite-merged image of FITC-stained anti-AVP and LRSC-stained anti-FGF2 immunoreactivities generated from 46 sequential 0.2 μm slices at 200X. Co-localization of FITC-labeled AVP and LRSC-labeled FGF2 immunoreactivities was confirmed by yellow fluorescence. Yellow fluorescence occurs in varying degrees in nearly all magnocellular neurons (arrowheads). Some red LRSC-stained structures lacking yellow fluorescence are probably astrocytes with processes (arrow).

Immunoreactive FGF2 and AVP were co-localized in the PVN of three human hypothalami. FGF2 immunoreactivity was found within the magnocellular neurons and their processes (Fig. 2A, arrowheads) which extended to the median eminence and posterior pituitary gland (not shown). FGF2 immunoreactivity was also clearly evident in endothelial cells within cerebral vessels (Fig. 2A, arrows) and astrocytes identified morphologically. Single labeled anti-AVP (FITC-stained) was detected within the neuronal perikarya and processes (Fig. 2B, arrowheads) of the neurons. AVP immunoreactivity was homogeneously distributed in the neuronal cytoplasm and processes. Neuronal nuclei were devoid of staining.

We demonstrated co-localized FGF2 and AVP immunoreactivity by the appearance of yellow fluorescence when the images of FITC-stained anti-AVP and LRSC-stained anti-FGF2 immunoreactivities were merged (Fig. 2C). Yellow fluorescence was seen in nearly all magnocellular neurons. Juxtaposed neuronal processes appeared as small yellow dots on the surface of cerebral vessels. Some of the red LRSC-stained structures, which lacked yellow fluorescence, were astrocytes and their processes.

### FGF2 and AVP immunoreactivity in human supraoptic nucleus

**Figure 3:**
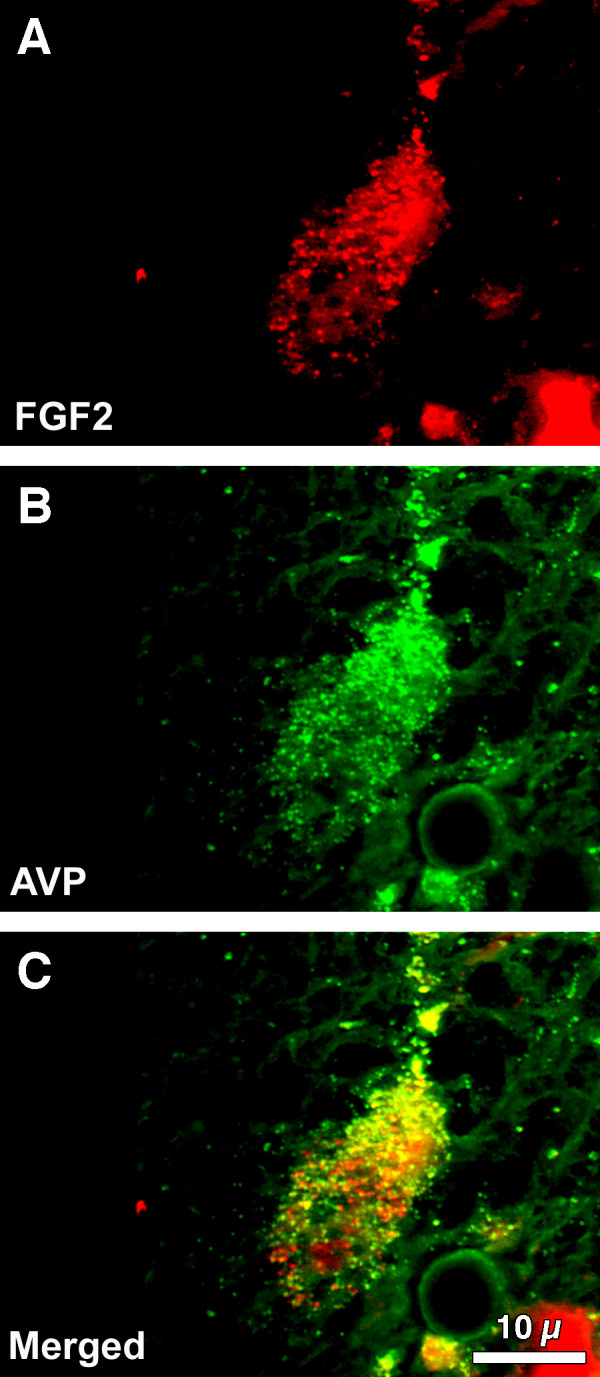
**Co-localization of FGF2 and AVP in human magnocellular neurons: **(A) Single labeled LRSC-stained anti-FGF2 and (B) FITC-stained anti-AVP images generated from 25 and 26 sequential 0.1 μm slices at 1000X magnification, respectively. Note differences in the relative distribution of reaction product within this neuron of the human SON. FGF2 is concentrated in small granular structures in the apical portion of the neuronal perikaryon (A). AVP, in contrast, is more homogeneously distributed in neuronal cytoplasm. (C) Composite-merged view of LRSC-labeled anti-FGF2 and FITC-labeled anti-AVP immunoreactivities generated from 51 sequential 0.1 μm slices at 1000X. Note the tendency for co-localized yellow reaction products to be concentrated in the apical portion of this neuronal cell body.

In the human SON, immunoreactive FGF2 was observed as small granule-like structures located primarily in the apical portion of the neuronal perikaryon, an area immediately above the nucleus of the neuron in which the Golgi apparatus and endoplasmic reticulum are located (Fig. 3A). In contrast, immunoreactive AVP was more homogeneously distributed throughout neuronal cytoplasm (Fig. 3B). Differential localization was more evident after merging LRSC-labeled anti-FGF2 and FITC-labeled anti-AVP immunoreactivities (Fig. 3C). Co-localized yellow immunofluorescence was concentrated apically in the neuronal cell body. In frequent observations, the juxtaposed neuronal processes appeared as small yellow dots on the surface of cerebral vessels (Fig. 3C, bottom).

### High resolution localization of FGF2 immunoreactivity in magnocellular neurons of human PVN

**Figure 4:**
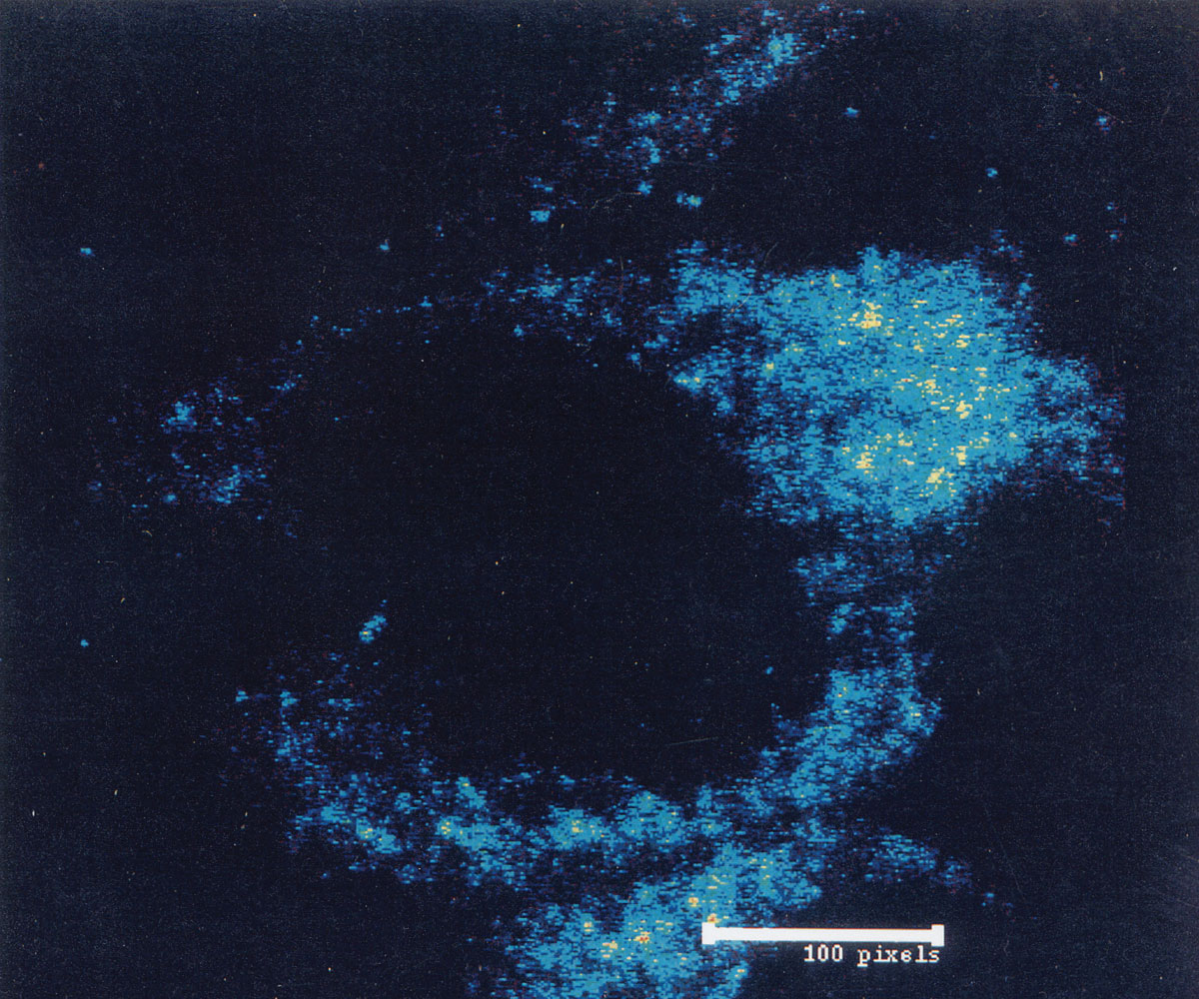
**FGF2 in granular structures within neurons in the human paraventricular nucleus (PVN): **High resolution (2000X) rendering of the distribution of LRSC-labeled anti-FGF2 immunoreactivity within the perikaryon of a neuron from human PVN generated with the full pixel array of 40 sequential 0.2 μm slices. The blue dots represent FGF2 immunoreactivity. The green and yellow dots indicate progressively greater anti-FGF2 reaction product density (green < yellow), which is most notable in the apical portion of the neuronal cytoplasm.

To delineate cytoplasmic distribution of FGF2 immunofluorescence, we examined LRSC-labeled anti-FGF2 immunoreactivity within the perikaryon of a neuron from human PVN at high resolution, using the full pixel array from 40 sequential 0.2 μm slices (Fig. 4). FGF2 reaction product was concentrated in small granule-like structures within apical cytoplasm. Staining was absent inside the nucleus, although some staining appeared on the nuclear surface.

### Immunolocalization of immunoreactive FGF2 and AVP in rat paraventricular nucleus

**Figure 5:**
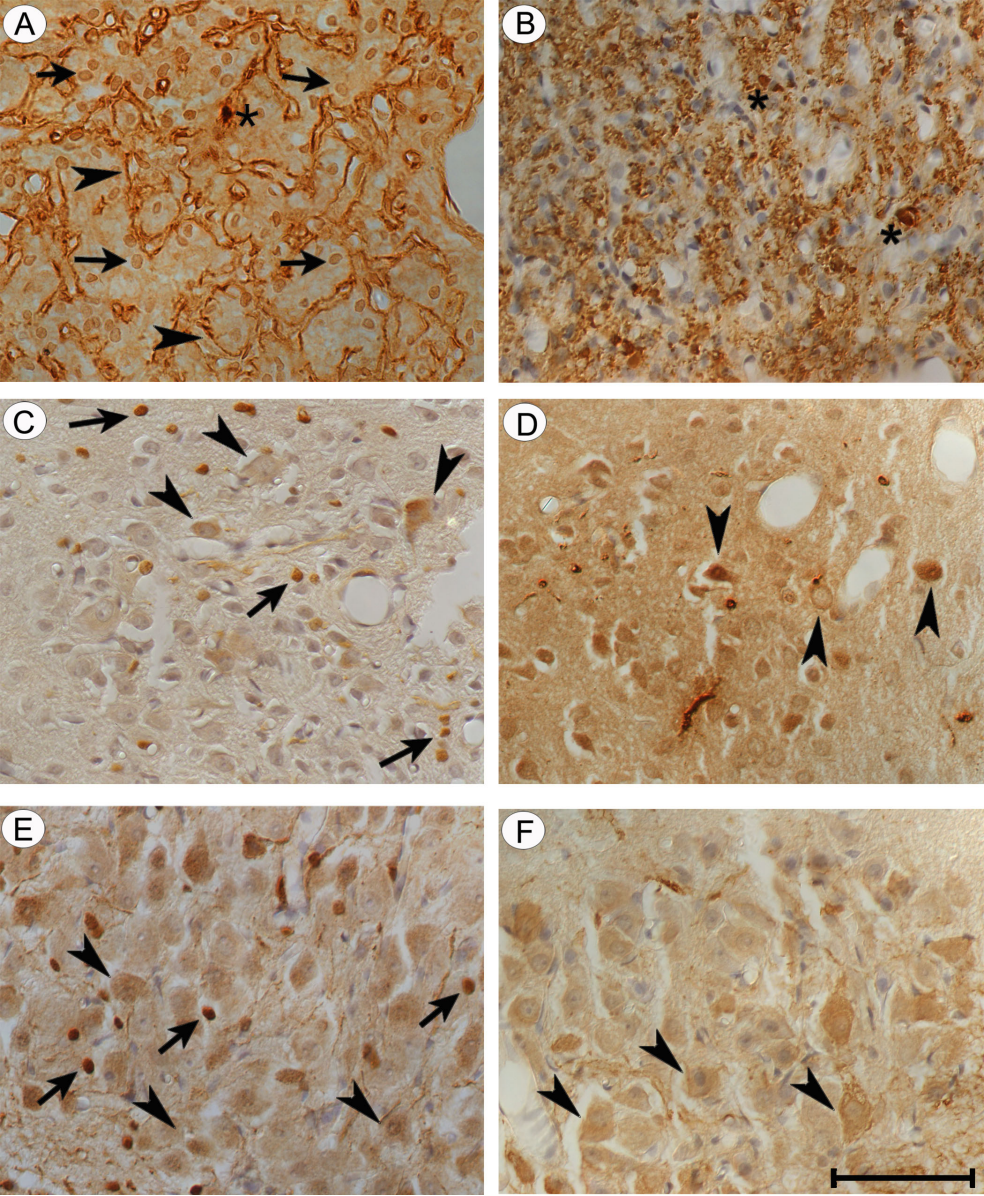
**Immunolocalization of FGF2 and AVP in hypothalamic and hypophyseal regions of adult rats: **(A) In the neural lobe, FGF2 is localized to basement membranes (arrowheads) and astrocytes (arrows), (B) whereas AVP is found in axonal fibers (stars) and Herring bodies (arrowheads). (C) PVN astrocytes (arrows) and neurons (arrowheads) appear to contain FGF2. (D) It is not clear if the same neurons express both FGF2 and AVP. (E) Most AVP-positive SON neurons (F) (arrowheads) are also stained for FGF2 (E). (Magnification bar = 100 μm)

In untreated rats, AVP as well as FGF2 immunolocalized in both the neurohypophysis and hypothalamus. Fig. 5 depicts FGF2 and AVP immunoreactivity in the normal neural lobe and hypothalamic nuclei. Although the neural lobe, PVN and SON all contained both peptides, there were significant regional differences in immunoreactive FGF2 distribution. In the neural lobe, FGF2 immunoreactivity mainly localized to pituicytes and basement membranes whereas immunoreactive AVP was mainly associated with Herring bodies and axonal fibers. In rat PVN, the astrocytes and parvocellular neurons contained FGF2 immunoreactivity. In the rat SON, astrocytes and selected populations of magnocellular neurons showed strong FGF2 immunostaining; however, we did not determine if the same neurons express FGF2 and AVP immunoreactivity Astrocytes were identified based on the characteristic nuclear immunostaining for FGF2 [14]. Neurons were identified based on their typical large round nucleus and central nucleolus. No staining was observed when sections were incubated with the antibody in the presence of the antigenic peptide or the eluant from the affinity column used to purify the antibody (not shown).

### Effects of chronic dehydration on the distribution and expression level of FGF2 in rats

**Figure 6:**
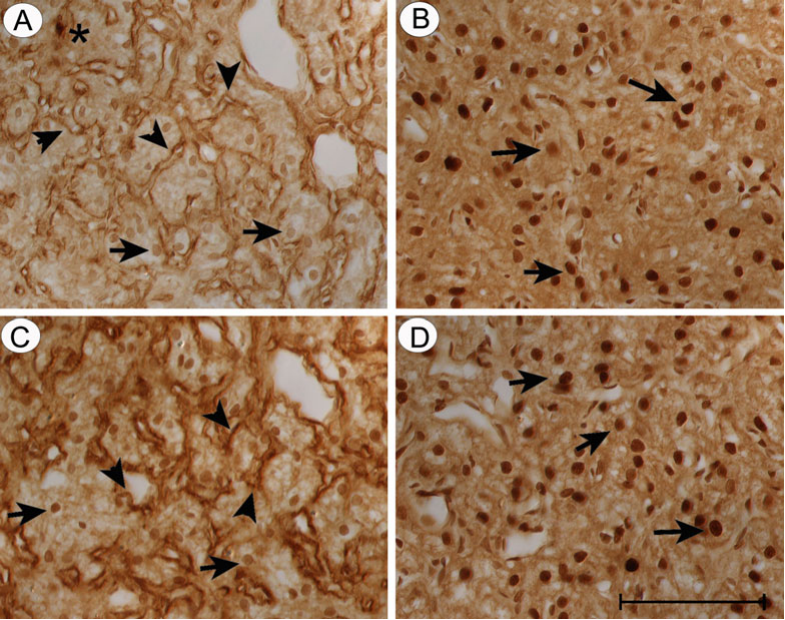
**Altered distribution of FGF2 in the rat neurohypophysis after dehydration: **In normal (A & B) and water-deprived rats (C & D), tissues were immunostained with polyclonal antibodies against FGF2: To visualize the changes in the localization of FGF2 in the extracellular matrix, tissue sections were treated with antibody Ab773 (A & C). To visualize the changes in intracellular FGF2, tissue sections were treated with antibody Ab106 (B & D). In control animals, FGF2 is associated with basement membranes (arrowheads), Herring bodies of axons of neurosecretory neurons (stars) (A) and pituicytes (arrows). Notice the characteristic beaded appearance of axons (B). In neurohypophyseal tissue from experimental rats, (C) and (D) show that the characteristic morphological changes associated with chronic dehydration are accompanied by increased FGF2 staining in structures underlying the perivascular space (arrowheads) (C). The hypertrophic pituicytes (arrows) also display strong nuclear staining (D). (Magnification bar = 100 μm)

To investigate FGF2's possible role in modulating water balance, we studied the localization and extent of immunoreactive FGF2 distribution in the neurohypophysis of normal and water-deprived rats. In control pituitary glands, FGF2 immunoreactivity localized to the neurohypophysis, pars distalis and pars intermedia. In neurohypophysis, immunoreactive FGF2 was associated with basement membranes, Herring bodies derived from magnocellular neurons (Fig. 6A) and pituicytes (Fig. 6B). It is interesting to note strong localization of FGF2 in the nuclear compartment of pituicytes. In the case of AVP, large number of positive Herring bodies was observed throughout the lobe, while fewer Herring bodies were intensely stained for FGF2. After 72 h of water deprivation, characteristic morphological changes were observed throughout the neural lobe. Extracellularly, there was a significant increase in the vascular basement membranes associated with the perivascular space (Fig. 6C). This was accompanied by an altered pattern of immunoreactive FGF2, i.e., the increased basement membrane structures within the perivascular space all stained for FGF2 (Fig. 6C, compared to Fig 6A). Scattered Herring bodies also displayed FGF2 immunoreactivity (Fig. 6D). Pituicytes displayed strong nuclear staining of FGF2 (Fig. 6D), although there was no significant increase in pituicyte cell numbers. Disrupted water balance thus resulted in enhanced expression of FGF2 in several regions of rat neurohypophysis. No staining was observed when sections were incubated with the antibody in the presence of the antigenic peptide or the eluant from the affinity column used to purify the antibody (not shown).

**Figure 7:**
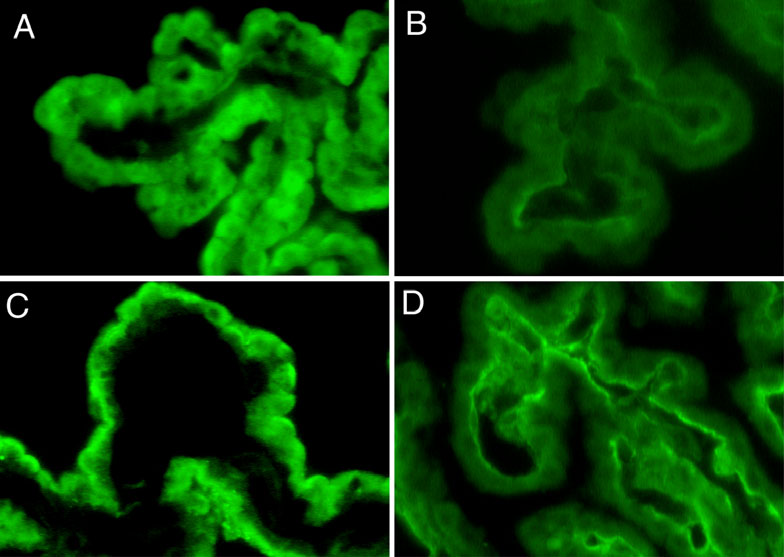
**Altered distribution of FGF2 in choroid plexus of rat brain after dehydration: **FGF2 immunostaining in lateral ventricle choroid plexus tissues was evaluated in adult rats exposed to chronic water deprivation for 72 h. Anatomically, the choroidal villus consists of a single layer of cuboidal epithelial cells that circumferentially surrounds an inner vascular core and extracellular matrix. The apical membrane faces the 'outside' CSF compartment; see Smith *et al. *[40] for a delineated description of choroid structural features. Note the marked FGF2 immunostaining in plexus parenchymal epithelium from a dehydrated animal (A), particularly in the nuclei, cytoplasm and the apical (CSF-facing) membrane, and the lower concentration in basement membranes (B). Comparatively, normal animals show lower levels of intracellular FGF2 in the epithelium (C), but stronger staining associated with basement membranes (D). Tissues were immunostained with polyclonal antibodies against FGF2: Ab106 (A & C) and Ab773 (B & D).

Choroid plexus epithelium of the lateral ventricle also displayed marked changes in the pattern of FGF2 immunostaining in animals dehydrated for 72 h. Following water deprivation, the levels of FGF2 immunoreactivity were augmented in the cytoplasm, apical membrane and nuclei of virtually all epithelial cells (Fig. 7A) but reduced in the extracellular matrix (Fig. 7B). This is in contrast to choroid tissues in control rats, with ad libitum water intake, in which epithelial cells showed low levels of intracellular FGF2 (Fig. 7C) but greater reaction product in basement membranes (Fig. 7D).

**Figure 8:**
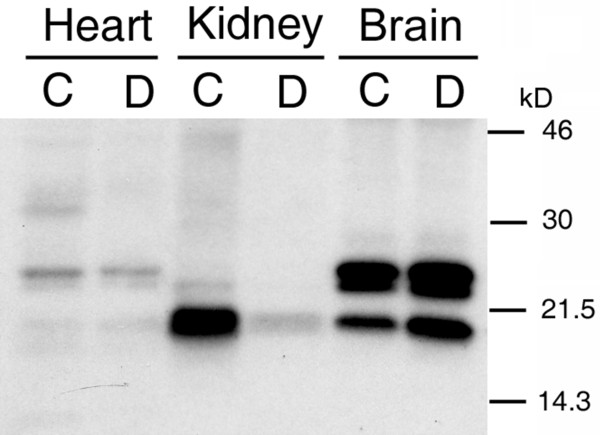
**Immunoblotting confirms enhanced hypothalamic FGF2 expression after dehydration: **FGF2 protein level was analyzed by western blotting for control (C) and dehydrated (D) states. Note the increased FGF2 levels in hypothalamus, resulting from 3 days of water deprivation, for the 3 isoforms of about 18, 23 and 24 kDa. Heart, kidney and brain rat tissues were extracted as described in the text and examined for rat FGF2 with a polyclonal antibody recognizing 3 forms of endogenous FGF2. Equal amounts of protein were loaded per well. Exposure time was 36 h. Dehydration specifically elevated the expression of FGF2 in hypothalamus but not in heart or kidney.

Immunoblotting was performed with hypothalamus, kidney and heart tissue extracts to determine if FGF2 isoforms were altered in dehydrated rats. Following water-deprivation for 3d, there was an increase (by western blot analysis) in the levels of all forms of FGF2 (ca. 18, 23 and 24 kDa) in the rat hypothalamus (Fig. 8). In contrast, in the kidney there was a clear reduction in the levels of FGF2 after dehydration but no changes were detected in the heart.

## Discussion

A co-localization of immunoreactive FGF2, AVP and their cognate receptors in the hypothalamus and choroid plexus epithelium [17-19] supports an hypothesized role [21] for integrating ion transport, membrane permeability and fluid balance in CNS. In peripheral tissues, the peptide trio of AVP, angiotensin II (AII) and atrial natriuretic peptide (ANP) maintain plasma volume and osmolality while FGF2 has significant hypotensive activity. By down-regulating CSF formation, the neuropeptides AVP, AII and ANP affect fluid homeostasis within the brain [1,5,7,8,16,27]; these same peptides also modulate fluid balance in peripheral tissues [28,29]. Both FGF2 and AII are linked to AVP release from cells that regulate CSF formation and blood flow in CNS. Accordingly, functional interactions among AVP, AII and FGF2 occur in choroid plexus, neuroendocrine regions and smooth muscle cells. FGF2, TGF-beta and other growth factors [30] help to regulate fluid balance by functionally coupling with fluid-regulating peptides (e.g. AVP and AII) both centrally and peripherally [31].

The study presented herein localized FGF2 in human and rat PVN and SON neurons, and in neurohypophysis. FGF2-like immunoreactivity was initially identified in hypothalamo-hypophyseal tissue by Iwata and colleagues [22] who described numerous immunoreactive neuronal processes originating from FGF2-positive cells extending lateroventrally and then caudally to the internal layer of median eminence. This pointed to a neuroendocrine-type pathway of FGF2 expression. In addition, the neurohypophysis contained many FGF2-like immunoreactive fibers. We extended Iwata's studies and, with confocal microscopy, determined that FGF2 immunoreactivity in the PVN and SON of these neuroendocrine-like pathways co-localizes with AVP.

At a subcellular level, the differential distribution of neuropeptides provides clues about function. By high-resolution analysis of hypothalamic neurons, we found that FGF2 immunostaining is associated with small granular structures at the apical portion of the perikaryon. Immunoreactive AVP, in contrast, appeared to be homogeneously distributed throughout neural cytoplasm. These localization patterns of immunoreactive FGF2 and AVP most certainly reflect different modes of peptidergic processing. Unlike AVP, the FGF2 peptide lacks a leader sequence that enables Golgi-mediated secretion. Thus, FGF2 is exported from the neuroendocrine cell by a non-Golgi-associated pathway [25,32] with apparent linkage to the alpha subunit of Na^+^-K^+ ^ATPase [23]. Many facets of AVP secretion are well understood [33]. However, future work should ascertain whether FGF2 is released at the apex of the perikaryon or if it regulates the release and/or processing of the AVP pro-hormone, as it does with luteinizing hormone-releasing hormone [34]. It is interesting to note that FGF2 and vasopressin immunoreactivities do not overlap within all neurons. Some neurons continue to express only one peptide or the other. Since the physiological mechanisms through which these two peptides interact in regulating fluid balance remain unknown, one can only speculate that the relative absence in some neurons may have a physiological significance that is yet to be determined.

In 1991, Frautschy *et al. *first hypothesized that FGF2 could be associated with water balance [21]. Since then, numerous studies have intimated an FGF2 involvement with hypophyseal-integrated water homeostasis [19,23,24]. The results presented herein are novel in describing that the hypothalamic-pituitary distribution of immunoreactive FGF2 in humans resembles that of AVP; and that dynamic changes in FGF2 expression occur in PVN, SON and choroid plexus in response to fluid dyshomeostasis. FGF2, as AII, likely promotes the release of cellular AVP, at least in choroid plexus; this leads to reduced CSF formation rate. We propose that the wide spectrum of actions of FGF2 in mitosis, angiogenesis and cell growth can now be extended to include a fluid-regulatory role [16,17].

To evaluate the putative role of FGF2 in water balance, we began by evaluating whether conditions that change water dynamics consequently alter the distribution and level of FGF2. We found prominent tissue remodeling and increased FGF2 immunoreactivity in the pituitary neural lobe in rats subjected to 72 h of water deprivation. Moreover chronic dehydration, salt loading and hypernatremia also characteristically upregulate AVP expression in the hypothalamic-pituitary axis [35] and choroid plexus [36]. Interestingly, a similar FGF2-AVP interaction has been previously noted during myocardial remodeling [37]. These multiple, connected observations lead us to think that the observed increase in neurohypophysial FGF2 is linked to augmented release of AVP from the pituitary during dehydration and/or elevated plasma osmolality [38]. Pituitary FGF2 up-regulation after water deprivation provides strong correlative evidence for a functional role of FGF2 in fluid balance because AVP, which co-localizes with FGF2 in the hypothalamic-pituitary axis and choroidal epithelium, regulates ion and water fluxes in V2- and V1-receptor-bearing targets such as kidney and choroid plexus [8].

It is fascinating that 3 isoforms of hypothalamic FGF2 were augmented by the 3-day dehydration (Fig. 8). On the other hand, enhanced expression of FGF2 in response to dehydration was not observed in the non-neural tissues compared: heart and kidney. Thus, in brain but not in cardiac and renal tissues, there was uniquely homeostatic-enhanced expression of all three known molecular weight forms (18, 23 and 24 kDa) of FGF2 (Fig. 8). These western blot data for FGF2 protein are consistent with the increased FGF2 immunostaining in SON and PVN (and choroidal epithelium) following water deprivation. Cumulative evidence therefore indicates that brain, which is responsible for regulating AVP-sensitive fluid balance via central (choroid plexus) and peripheral (kidney) organs, homeostatically modulates its FGF2 and associated AVP levels in the face of dehydration. It is interesting to note the decreased levels of FGF2 in the kidney after chronic dehydration taking into account that peripheral water homeostasis is regulated by hypothalamic AVP. The data presented here and the previous reports on the increased levels of FGF-2 mRNA in the hypothalamus after fasting [39] suggest that FGF2 may play a general role in regulating hypothalamic function under different stress conditions.

## Conclusions

Taken together, the analyses described herein for human and rat CNS tissues, in conjunction with previous findings localizing FGF2 and AVP peptides and their receptors (involving structures associated with fluid homeostatic functions), support the hypothesis that FGF2 participates in water balance by modulating responsive cells in the posterior pituitary and choroid plexus. In light of the dehydration response and the substantial increase of FGF2 in neuroendocrine-type cells such as neurohypophyseal and choroid epithelial cells that also contain AVP, the findings strengthen the working model that the endocrine control of fluid transport and membrane permeability may involve locally-acting paracrine factors such as FGF2 in the CNS. Whether these apparent linkages among FGF2, AVP and water balance exist in peripheral tissues is under investigation.

## Abbreviations

AII: angiotensin II; ANP: atrial natriuretic peptide; AVP: arginine vasopressin; BSA: bovine serum albumin; CNS: central nervous system; DAB: diaminobenzidine; FGF2: basic fibroblast growth factor; FGFR: receptor for basic fibroblast growth factor; PBS: phosphate-buffered saline; PVN: paraventricular nucleus; SON: supraoptic nucleus; TGF beta: transforming growth factor beta; V1 and V2: subtypes of receptor for AVP

## Competing interests

The authors declare that they have no competing interests.

## Authors' contributions

AMG: Designed and performed rat experiments, data analysis and interpretation, manuscript drafting and revision. WMT: Performed confocal microscopy of the human brain tissues. CEJ: Designed *in vivo *studies, supervised critical data discussion, and coordinated manuscript drafting and revision. JCK: Directed and interpreted the confocal analyses of human hypothalamus specimens. WEL: Performed rat experimentation, data analysis, and manuscript drafting. EGS: Designed human hypothalamus/neurohypophysis studies, data analysis and interpretation, manuscript revision. AB: Designed rat experiments, critical data discussion and manuscript revision.

All authors have read and approved the final version of the manuscript
